# Protein tyrosine phosphatases: new markers and targets in oncology?

**DOI:** 10.3747/co.2008.195

**Published:** 2008-01

**Authors:** S. Hardy, M.L. Tremblay

**Affiliations:** * The McGill Cancer Center and the Department of Biochemistry, McGill University, Montreal, QC

**Keywords:** Protein tyrosine phosphatases, tumour suppressor, oncogene, cancer treatment, pharmacologic inhibitor, prognostic marker

## 1. INTRODUCTION

The discovery nearly 30 years ago that v-*src* (the form of the Src kinase encoded by Rous sarcoma virus) employs tyrosine kinase activity for transforming cells led, decades later, to a revolution in molecular medicine. The identification of key signalling pathways controlled by this reversible phosphorylation opened a new means by which cell machinery could be activated or inhibited. As these signalling pathways were discovered to contribute in multiple ways to the oncogenic process, tyrosine kinases became key targets for clinical interventions. Today, it is routine to see cancer treatment regimens that include the use of kinase inhibitors such as anti-receptor antibodies or small molecules. Some examples include Herceptin (Genentech, San Francisco, CA, U.S.A.), Gleevec (Novartis Pharmaceuticals, St. Louis, MO, U.S.A.), Iressa (AstraZeneca Pharmaceuticals, Wilmington, DE, U.S.A.), and Tarceva (Genentech)^1^–^4^.

Interestingly, it took more than 10 years after recognition of the protein-tyrosine kinases (ptks) for the first member of the family of protein-tyrosine phosphatases (ptps) to be identified^5^. The fact that the ptps have no structural similarity to serine threonine phosphatase may have contributed to the difficulties encountered in cloning those enzymes.

Because the ptps perform enzymatic reactions that are the reverse of those performed by the ptks, the ptps were assumed to be tumour suppressors. Enzymes such as pten that counteract the activation of phosphoinositide-3-kinase (pi3k) are an excellent example of such functioning by a ptp^6^. However, more recently, several findings have acknowledged that, in many circumstances, members of the ptp superfamily act as typical oncogenes^7^. Recent advances in the generation of ptp inhibitors and positive outcomes in clinical trials of *PTPN1* (protein tyrosine phosphatase, non-receptor type 1; formerly *PTP1B*) antisense for treatment of type 2 diabetes may soon lead to the use of ptps as markers and novel targets in oncology.

In the present paper, we comment on the diversity of the ptp superfamily, describe three of the main members that are candidates with the most interesting potential for cancer treatments, and briefly survey the major hurdle standing in the way of the development of small-molecule ptp inhibitors for clinical applications.

## 2. THE PTP SUPERFAMILY

Reversible tyrosine phosphorylation regulates many cellular functions, including cell proliferation, survival, adhesion, and migration^8^. In contrast to the ptks, which phosphorylate their substrates on tyrosine residues, members of the ptp family remove phosphate from protein and from phospholipid substrates. The ptps are now recognized to constitute a large, structurally diverse family of tightly regulated enzymes with important regulatory roles^8^. Recently, Alonso *et al.*^9^ identified a total of 107 ptps encoded in the human genome. This superfamily of ptps can be divided into four major enzyme subfamilies. Enzymes from three of those families (classes i–iii) contain a catalytic oxidation-sensitive active cysteine residue embedded within a conserved signature motif (XH**C**SXGXGRXG), which carries out catalysis by executing a nucleophilic attack on its phospho substrate^10^.

Class i is the largest ptp subfamily, comprising the 38 classical ptps and 61 dual-specificity phosphatases. Classical ptps show specificity for phosphotyrosine, and the dual-specificity phosphatases can dephosphorylate phosphotyrosine-, phosphoserine-, and phosphothreonine-containing substrates. Among the class i members, pten and the myotubularin subfamily are the enzymes that preferentially target phospholipids. Class ii has a single member, the so-called low molecular weight ptp (lm-ptp), and class iii encompasses the three mammalian Cdc25 proteins, which participate in cell-cycle regulation. Class iv is a newly identified aspartate-based ptp family related to the Eya family.

### 2.1 *PTEN*: A KEY TUMOUR SUPPRESSOR IN CANCERS

The *PTEN* (phosphatase and tensin homologue) gene on chromosome 10q23.31, also known as *MMAC1* (mutated in multiple advanced cancers), encodes a tumour-suppressor protein that acts as an inhibitor of the pi3k-akt pathway. By removing the 3′ phosphate group of phosphatidylinositol-(3,4,5)-triphosphate, pten counteracts the activity of pi3k in promoting cellular growth, survival, and angiogenesis^6^. Mutations in *PTEN* are responsible for Cowden syndrome and other autosomal dominant disorders such as Bannayan–Riley–Ruvacalba syndrome and the Proteus and Proteus-like syndromes^11^.

Apart from its role in hereditary syndromes, pten inactivation has been identified in a series of sporadic human cancers, including glioblastomas of the central nervous system, endometrial carcinoma, prostatic adenocarcinoma, and melanoma^12^,^13^. Interestingly, some of the *PTEN* mutations may lead to protein instability and rapid removal through protein ubiquitination^14^. As a therapeutic strategy for *PTEN-*related cancer, it may perhaps be possible to target the specific ubiquitin ligase involved in that process, thus blocking pten degradation and maintaining, or restoring, some phosphatase activity that could prevent akt pathway activation.

### 2.2 Shp2 and Leukemias

Shp2 is encoded by the *PTPN11* gene in humans. It transduces mitogenic and pro-migratory signals from various receptor types via activation of the *ras*/erk cascade^15^.

Somatic mutations of *PTPN11* are the major cause of sporadic juvenile myelomonocytic leukemia, accounting for about 35% of cases^16^. In addition, mutations of this gene also occur in approximately 6% of patients with childhood acute lymphoblastic leukemia^17^ and in 4%–5% of patients with acute myeloid leukemia^18^. Mutations of *PTPN11* are uncommon in solid human tumours, but they have been detected in neuroblastoma, melanoma, lung adenocarcinomas, and colon cancer^18^.

Most of the mutations in the *PTPN11* gene lead to expression of Shp2 variants with single aminoacid changes that enhance the protein’s activity. In addition to its well-established pro-oncogenic potential in various types of leukemia, Shp2 is also a key downstream target of other oncogenes shown to drive excessive mammary epithelial cell proliferation and a potentiator of *neu-*induced transformation in mouse models^19^,^20^. Inhibiting Shp2 thus offers several new therapeutic avenues in several types of cancer.

### 2.3 *PTPN1*: A Surprising Oncogene

As the prototype for the superfamily of ptps, ptpn1 has been implicated in multiple signalling pathways, including pathways triggered by growth factors, hormones, and cytokines^21^. Because it is a negative regulator of oncogenic tyrosine kinase receptors such as the insulin receptor and insulin-like growth factor 1 receptor, ptpn1 was first thought to be a tumour suppressor. Yet surprisingly, *PTPN1* knockout mice do not develop cancers.

Interestingly, *PTPN1* is located within 20q13, a region frequently amplified in ovarian and breast cancers and usually associated with a poor prognosis^22^. Immunocytochemical studies have shown that *PTPN1* is overexpressed in 40% of human breast cancers^23^,^24^, and furthermore, ptpn1 has been shown to be able to activate the oncogene *src*^25^ and has been reported to have a positive role in the *ras* pathway^26^.

Recently, our group and Dr. Benjamin Neel’s laboratory demonstrated that deletion of *PTPN1* activity in mmtv-*neu* transgenic mice by breeding with *PTPN1*-deficient mice caused significant mammary tumour latency and resistance to lung metastasis^27^,^28^. Our group also used a specific *PTPN1* inhibitor that protected the mmtv-*neu* transgenic mice from developing tumours to confirm those findings^27^. The latter study also demonstrated that *PTPN1* is a true oncogene, because specific overexpression of *PTPN1* in the mammary gland led to the development of spontaneous breast cancer^27^. Therefore *PTPN1,* previously recognized for its role in downregulating insulin signalling, has now been shown to function as a positive regulator of signalling events associated with breast tumorigenesis.

## 3. PHARMACOLOGIC TREATMENT OF *PTPN1*

Several years ago, our group, in collaboration with Brian Kennedy of Merck–Frosst, confirmed that *PTPN1* inhibition could have a significant benefit in type 2 diabetes^29^. That finding triggered much research into the development of small molecules against *PTPN1.*

Unfortunately, the close similarities between the various members of the ptp family and the general hydrophilicity of small molecules that bind to the ptp active pocket remain major hurdles in the development of specific inhibitors against *PTPN1*. It appears that the ptp field has now reached the stage occupied by the kinase drug development field more than 10 years ago. For example, Isis Pharmaceuticals developed and, in late phase ii clinical trials, successfully employed *PTPN1* antisense inhibitors for the treatment of type 2 diabetes^30^. Their study clearly validates *PTPN1* as a safe target in humans. In that context, the race for small-molecule inhibitors will certainly gain momentum. Other companies should soon emerge with their own versions of anti-*PTPN1* small drugs. It stands to reason that these inhibitors should rapidly be tested in cancers in which *PTPN1* is overexpressed.

## 4. SUMMARY

It is now clear that ptps have both inhibitory and stimulatory effects on cancer-associated signalling processes, and depending on their associated proteins and substrates, they act as oncogenes in multiple human cancers. Soon, several ongoing studies should validate ptps such as pten and ptpn1 as useful prognostic markers and potentially novel targets in cancer therapies.

Although much current interest surrounds the clinical introduction of specific ptk inhibitors, chemical targeting of ptps remains largely unexplored. Despite major efforts by the pharmaceutical industry (given that these targets were identified more than 10 years after the tyrosine kinases), the development of small-molecule inhibitors of ptps is still in its early stages. Phosphatases represent 4% of the “drug-able” human genome^31^, and the rapidly increasing number of human diseases associated with ptp abnormalities—including cancer—has begun to elicit a growing interest in ptps as drug targets in oncology. The recent identification of *PTPN1* as a potential oncogene in breast cancer may be key in focusing research efforts toward this relatively poorly known gene family.
